# Peaceful acceptance and struggle with terminal cancer: The role of mindfulness, self-compassion, and body image distress

**DOI:** 10.1017/S1478951525000094

**Published:** 2025-03-14

**Authors:** Francesco De Vincenzo, Rossella Mattea Quinto, Luca Iani, Sieva Durante, Chiara Scalpelli, Luigi Lombardo

**Affiliations:** 1 Department of Human Sciences, European University of Rome, Rome, Italy; 2 U.O. di Cure Palliative, Fondazione Sanità e Ricerca, Rome, Italy

**Keywords:** Body image, hospice, end of life, terminally ill, palliative care

## Abstract

**Objectives:**

This study aimed to examine the extent to which mindfulness, self-compassion, and body image distress are associated with peaceful acceptance or struggle with illness in terminally ill cancer patients, after controlling for psychological distress, sociodemographic characteristics (age, gender, education, marital status), and clinical characteristics (body mass index, Karnofsky Performance Status, time since diagnosis).

**Methods:**

A cross-sectional study was conducted with 135 terminally ill cancer patients. Participants were consecutively sampled. Two five-step hierarchical regression models were performed, one for peaceful acceptance and the other for struggle with illness. The models included sociodemographic (step 1), clinical characteristics (step 2), psychological distress (step 3), mindfulness and self-compassion (step 4), and body image distress (step 5).

**Results:**

Body image distress was negatively associated with peaceful acceptance after controlling for the other variables. Both body image distress and self-compassion were uniquely associated with struggle with illness, in a positive and negative direction, respectively. The overall models explained 33% of the variance in peaceful acceptance and 61% in struggle with illness.

**Significance of results:**

Targeting body image distress may be important for both enhancing peaceful acceptance and reducing struggle with one’s terminal condition. Addressing self-compassion, however, may help patients alleviate the struggle alone. These findings suggest that peaceful acceptance and struggle with illness may follow different clinical pathways with partly different underlying mechanisms. This study provides a foundation for future research to develop interventions for body image and self-compassion specifically tailored to the needs of terminally ill cancer patients.

## Introduction

The Lancet Commission on the Value of Death emphasized the need for a new perspective on end-of-life (EOL) care, proposing principles that advocate for a new vision on death and dying (Sallnow et al. 2022). One of these principles reframes death as a relational and spiritual process rather than just a physiological event. Within this framework, accepting one’s terminal condition is an integral part of the EOL care system (Bhadelia et al. 2022; Sallnow et al. 2022; Zimmermann 2012).

Acceptance in EOL care has been conceptualized in terms of cognitive and emotional preparedness for death. Cognitive preparedness involves one’s disease progression (i.e., prognostic awareness), while emotional preparedness refers to the emotional acceptance of one’s terminal condition (Tang et al. 2019, 2020; Wen et al. 2022, 2021, 2023). According to Kübler-Ross’ stages of dying, acceptance is viewed as possible and desirable, always counterpoised to denial (Kübler-Ross 1969; Zimmermann 2012). In this framework, acceptance essentially signifies *emotional* acceptance of impending death, representing “emotional equanimity – a sense of inner peace and tranquillity that comes with the letting go of a struggle to regain what is lost or being taken away” (Prigerson and Maciejewski 2008, p. 435). Prognostic awareness and emotional acceptance are distinct yet related phenomena (Mack et al. 2008; Prigerson and Maciejewski 2008; Ray et al. 2006; Tang et al. 2020; Wen et al. 2021). Terminally ill cancer patients not achieving conjoint cognitive and emotional acceptance, or cognitive acceptance only, experienced higher levels of anxiety, depression, and a worse quality of life, compared to those who achieved both (Wen et al. 2022). Interestingly, there were no differences between those in a conjoint state of acceptance and those reporting emotional acceptance only (Wen et al. 2022), possibly suggesting that emotional acceptance may play a more crucial role in psychological functioning.

Emotional acceptance can be further delineated into two dimensions, namely *peaceful acceptance* and *struggle with illness* (Mack et al. 2008; Prigerson and Maciejewski 2008). While some patients approach terminal illness with integrity, by finding meaning and maintaining dignity, others experience despair (Mack et al. 2008). The former exhibit calmness, peace, and equanimity (i.e., peaceful acceptance), while the latter manifest feelings of foreboding, fear, anger, rage, injustice at their terminal condition (i.e., struggle with illness; Mack et al. 2008; Okamura et al. 2022). Lower peaceful acceptance and higher struggle with illness have been associated with psychopathology, severe anxiety and depressive symptoms, worse physical, emotional, functional, and spiritual well-being (Mack et al. 2008; Okamura et al. 2022).

Despite the importance of emotional acceptance, few studies explored its psychological and social predictors. Higher social support, lower patient-family communication about EOL issues, and spiritual well-being were associated with emotional acceptance (i.e., emotional preparedness; Wen et al. 2023; Wentlandt et al. 2012). Moreover, to the best of our knowledge, no previous study examined the predictors of both peaceful acceptance and struggle with illness. Emanuel et al. (2007) identified three key elements of the dying role: practical, relational, and personal, with the latter involving tasks that promote personal growth and the finishing of one’s life story (e.g., adjustment to loss, existential tasks). We propose that mindfulness and self-compassion may represent positive resources which foster the personal element of the dying role, while body image distress may act as a hindering factor.

Mindfulness involves attentional awareness and a non-judgmental stance towards experiences (Baer et al. 2006; Bohlmeijer et al. 2011; Kabat-Zinn 1990). Self-compassion involves being open to one’s own suffering and generating the desire to alleviate it with kindness (Neff 2015). While mindfulness and self-compassion share similarities, self-compassion uniquely emphasizes self-kindness and the recognition of suffering as a shared human experience (Neff and Dahm 2015). Moreover, the mindfulness component of self-compassion specifically refers to the awareness of negative experiences, rather than all valenced experiences (e.g., positive, neutral; Neff and Dahm 2015). In non-terminal cancer patients, mindfulness and self-compassion have been associated with improvements in quality of life, anxiety and depressive symptoms, and spiritual well-being (Garcia et al. 2021; Stadnyk et al. 2024; Torricelli et al. 2023; Zimmermann et al. 2018). Within the EOL context, studies focused almost solely on formal and informal caregivers (Conversano et al. 2020; Covington et al. 2023). Nonetheless, a qualitative study with terminally ill cancer patients found several components that facilitate a mindful living at the EOL, including a purposeful examination of inner experiences related to illness and mortality (Choo et al. 2024).

Body image distress is a significant concern for cancer patients, affecting emotional and physical well-being (Esplen and Fingeret 2021) across various stages of the cancer patients’ trajectory (Ivanova et al. 2023; Melissant et al. 2021; Nikita Rani and Kumar 2022; Paterson et al. 2016; Sebri et al. 2024), including palliative care (Diaz-Frutos et al. 2016). Although research on body image has mainly focused on early-stage cancers, evidence suggests that it remain influential even for those with a shortened life expectancy (McClelland et al. 2015; Vas et al. 2019). For metastatic breast cancer patients, body image was associated with physical and emotional functioning (McClelland et al. 2015). Moreover, appearance-focused struggles and low body confidence were two of the most disturbing issues reported by palliative care outpatients (Vas et al. 2019). Notably, body image distress stemmed from patients’ “frustration over their lack of control and their attachment to their former self-image” (Vas et al. 2019, p. 6). This distress may reflect a key aspect of the personal element of the dying role, where patients must adjust their sense of self in response to physical changes, integrating these losses into their evolving identity (Emanuel et al. 2007).

The objective of the present study was to explore how mindfulness, self-compassion, and body image distress are associated with peaceful acceptance and/or struggle with illness in terminally ill cancer patients. According to positive clinical psychology, positive characteristics can uniquely predict clinical outcomes beyond the predictive power of negative characteristics (e.g., psychological distress; Wood and Tarrier 2010). Thus, we hypothesized that mindfulness and self-compassion would be positively associated with acceptance and negatively with struggle after controlling for psychological distress. We further hypothesized that self-compassion would show stronger associations with these outcomes compared to mindfulness: self-compassion may be particularly beneficial in times of suffering, since it focuses on painful experiences, common humanity, and self-soothing, which is supported by previous studies showing that self-compassion was a stronger predictor of positive and negative functioning rather than mindfulness alone (Neff and Dahm 2015; Svendsen et al. 2020). Drawing upon previous studies indicating the relevance of body image and attachment to one’s own former self-image in palliative care (McClelland et al. 2015; Vas et al. 2019), it was also hypothesized that body image distress would be associated with lower acceptance and higher struggle. Finally, we aimed to identify potential psychological factors that may differentially influence acceptance and struggle.

## Methods

### Study design and sample

We conducted a cross-sectional study involving 135 terminally ill cancer patients receiving palliative care. Participants were consecutively enrolled at the Palliative Care Unit of the Hospice Fondazione Sanità e Ricerca, Rome, between January 2022 and March 2023, and were referred from hospitals, nursing homes, rest homes, and long-term care facilities. Inclusion criteria were age over 18, diagnosis of a life-threatening oncological disease with a prognosis ranging from 1 and 6 months (based on the evaluation of physicians referring the patients), the ability to read and speak Italian and provide written informed consent. Exclusion criteria were the presence of psychotic illness, dementia, severe neurological impairment, and a Karnofsky Performance Status (KPS) lower than 30, as these conditions could interfere with cognitive, emotional, and physical functioning, limiting reliable self-reporting and engagement in study assessments. A psycho-oncologist verified that patients met the eligibility criteria by reviewing clinical records. Eligible patients were then invited to participate, with the psycho-oncologist explaining the study’s aim and procedure and providing informed consent. This study was approved by the Ethics Committee of the European University of Rome (N. 01/2022) and complied with the Declaration of Helsinki.

### Measures

Eligible patients provided information on sociodemographic characteristics (i.e., age, sex, marital status, education level). Clinical data, including disease type, body mass index (BMI), KPS, time since diagnosis were collected from clinical records.

Dispositional mindfulness was measured with the short form of the Five Facet Mindfulness Questionnaire (Bohlmeijer et al. 2011). Participants rated the extent to which 24 items were true for them on a five-point scale (1 = *never or very rarely true*; 5 = *very often or always true*). A higher total score indicates a higher level of dispositional mindfulness. In this sample, the Cronbach’s α was .84.

Self-compassion was measured with the 12-item Self-Compassion Scale-Short Form (Raes et al. 2011). The items are rated on a five-point Likert scale from 1 (*almost never*) to 5 (*almost always*). Higher total scores reflect higher self-compassion. In this sample, reliability was acceptable (Cronbach’s α = .62).

Body image distress was measured with the Body Image Scale (BIS; Hopwood et al. 2001). The BIS is unidimensional and is composed by 10 items, which are rated on a 4-point Likert scale ranging from 0 (*not at all*) to 3 (*very much*). For the item 10, “*did you feel dissatisfied with the appearance of your scar?*,” the original BIS provides an alternative response “*not applicable*,” which is scored as 0. Since the BIS was originally developed and used in oncological populations rather than in terminally ill cancer patients (Melissant et al. 2018), some items may not be fully relevant for terminally ill cancer patients. To avoid forcing participants to provide responses that did not reflect their actual experiences, we added the “*not applicable*” response option to all items. Scores are summed to calculate the BIS total score, whereby “*not applicable*” answers were scored as 0. Higher scores on the BIS indicate higher body image distress. In the present study, the “*not applicable*” response was selected only for item 10 (“*did you feel dissatisfied with the appearance of your scar?*”) by 40 participants, and for item 6 (“*Have you been feeling less sexually attractive as a result of your disease or treatment?*”) by 4 participants. Item reliability was good (Cronbach’s α = .88).

The Peace, Equanimity and Acceptance in the Cancer Experience (PEACE; Mack et al. 2008) evaluates the degree of emotional acceptance of terminally ill conditions in patients with advanced cancer undergoing palliative care. The PEACE is composed by 12 items rated on a 4-point Likert Scale, from 1 (*not at all*) to 4 (*to a large extent*). It includes two subscales: Peaceful acceptance and Struggle with illness. The Peaceful acceptance subscale (5 items) evaluates the extent to which patients accept their terminal cancer and experience a sense of inner peace, equanimity, and harmony. Sample items include: “To what extent would you say you have a sense of inner peace and harmony?” and “To what extent do you feel that you have made peace with your illness?.” The Struggle with illness subscale (7 items) measures the extent to which patients struggle with their terminal illness, marked by feelings such as anger, rage, injustice, fear, and a sense of foreboding. Sample items include: “To what extent do you think your illness has beaten you down?” and “To what extent do you feel ashamed of, or embarrassed by, your current condition?.” Higher scores on both subscales indicate higher peaceful acceptance and greater struggle, respectively. The score ranges for the subscales are 5 to 20 for Peaceful acceptance and 7 to 28 for Struggle with illness. In the present study, the reliability of the subscales was good (Cronbach’s α for Peaceful acceptance = .81; Cronbach’s α for Struggle with Illness = .83).

Psychological distress was measured with the 4-item screening tool Patient Health Questionnaire-4 (PHQ-4; Kroenke et al. 2009). The PHQ-4 has two dimensions: anxiety and depression. All the items are rated on a 4-point Likert Scale, from 0 (*not at all*) to 3 (*nearly every day).* A higher total score indicates a higher level of psychological distress. In the current study, reliability was good (Cronbach’s α = .83).

### Data analysis

All statistical analyses were performed using IBM SPSS Windows (version 22.0). Occasional missing values were imputed by computing the mean score of the respective sub-scale for each participant. An *a priori* power analysis with GPower 3.1.9.7 (Faul and Erdfelder 1992) was based on a medium effect size (*f*^2^ = .15), with an α-error probability of .05, a statistical power (1 – *β* = 80 %), and the number of tested predictors and covariates (*k* = 11). The predictors included self-compassion, mindfulness, and body image distress, while the covariates were age, sex, education, marital status (1 = married; 2 = unmarried/widowed/divorced/single), time since diagnosis, KPS, BMI, and psychological distress. The analysis indicated that a sample size of at least 123 participants was required.

According to recommendations of Kim (2013) for medium-sized samples (50 < *n* < 300), variables were considered normally distributed if the absolute z-values of skewness and kurtosis were smaller than 3.29. Since this criterium was met, relationships between variable were examined using Pearson *r* correlation coefficients. To examine the extent to which the dependent variables (peaceful acceptance and struggle with illness) were accounted for by mindfulness, self-compassion, and body image distress, two five-step hierarchical linear regression analyses were carried out. In the first, the second, and the third step, covariates were added. Specifically, sociodemographic variables were entered in the first step (age, sex, education, and marital status). In the second step, clinical variables were added (KPS, BMI, time since diagnosis). In the third step, psychological distress was entered. The fourth and the fifth step were characterised by tested predictors. Mindfulness and self-compassion were added in the fourth step, while body image distress in the fifth step. All statistics were considered significant if *p* < 0.05.

Before running the analyses, assumptions were investigated for each statistical model. Multivariate outliers were investigated with Cook’s distance; values larger than 1.00 were considered outliers (Tabachnick et al. 2007). Normality of residuals was investigated through inspection of P-P plots (Field 2013) and by checking that absolute z-values of skewness and kurtosis were smaller than 3.29 (Kim 2013). No outliers were identified, and normality assumption was fulfilled. Multicollinearity was examined by computing tolerance values and Variance Inflation Factor (VIF), showing no interfering interactions between variables (tolerance values > .10 and VIF < 5). Linearity and homoscedasticity were assessed by plotting standardized residuals against predicted values. The scatterplot appeared to be horizontal in nature and fit a rectangular shape (Field 2013).

## Results

### Sample characteristics

A total of 148 patients were approached to participate in this study. Thirteen patients declined participation for the following reasons: unwillingness to sign the informed consent (N = 2), choosing not to participate (N = 9), incapacity to sign the informed consent (N = 2). Thus, a total of 135 terminally ill cancer patients consented and completed study procedures. Sociodemographic and clinical characteristics, as well as the mean levels of peaceful acceptance and struggle with illness are shown in Table 1. The mean age was 74.19, ranging from 45 to 94. Of all participants, 53.3% were female and 45.9% were married. The majority attended secondary school education or higher (54.8%). The most common diagnoses were lung (26.7%), colorectal (12.6%), breast (9.6%), and urological (9.6%) cancers. The average time since diagnosis was 31.10 months and 65.2% of participants had a KPS of 30. The mean level of peaceful acceptance was 12.61 (*SD* = 3.3), while the mean level of struggle with illness was 18.28 (*SD* = 4.6).

**Table 1.** Sample characteristics

Age, *M* (*SD*)	74.16 (11.945)
Gender, *N* (%)	
Female	72 (53.3)
Male	63 (46.7)
Marital status, *N* (%)	
Married	62 (45.9)
Unmarried/widowed/separated	73 (54.1)
Education, *N* (%)	
Elementary	25 (18.5)
Middle school	36 (26.7)
Secondary school	50 (37.0)
Degree	24 (17.8)
Cancer diagnosis, *N* (%)	
Lung	36 (26.7)
Breast	13 (9.6)
Brain	3 (2.2)
Colorectal	17 (12.6)
Gynecological	8 (5.9)
Oropharyngeal	5 (3.7)
Pancreatic	10 (7.4)
Urological	13 (9.6)
Hematopoietic/lymphoid	12 (8.9)
Thyroid	2 (1.5)
Gastrointestinal	4 (3.0)
Hepatic/biliary tract cancer	2 (1.5)
Melanoma	2 (1.5)
Kidney	2 (1.5)
Prostate	5 (3.7)
CUP	1 (0.7)
Months since diagnosis, *M* (*SD*)	31.10 (46.759)
KPS, *N* (%)	
30	88 (65.2)
40	34 (25.2)
50	13 (9.6)
Peaceful acceptance, *M* (*SD*)	12.61 (3.3)
Struggle with illness, *M* (*SD*)	18.28 (4.6)

CUP, cancer of unknown primary site; KPS, Karnofsky Performance Status.

### Correlation analyses

The results of Pearson correlations are reported in Table 2. The correlation between peaceful acceptance and struggle with illness was strong and negative. Struggle with illness was negatively correlated with mindfulness and self-compassion, and positively correlated with body image distress, with correlation coefficients ranging from moderate to strong. Peaceful acceptance was significantly and positively correlated with higher levels of mindfulness and self-compassion, while the correlation with body image distress was negative. The correlation coefficients were moderate in size.

**Table 2.** Correlation analyses

	1	2	3	4	5
1. Mindfulness	–				
2. Self-compassion	.491***	–			
3. Body image distress	−.221**	−.306***	–		
4. Peaceful acceptance	.423***	.391***	−.392***	–	
5. Struggle with illness	−.457***	−.538***	.670***	−.636***	–

* *p* < .05; **p < .01; ***p < .001.

### Factors associated with peaceful acceptance and struggle with illness

Hierarchical regression models were carried out to examine the ability of self-compassion, mindfulness, and body image to explain peaceful acceptance and struggle with illness, after controlling for sociodemographic and clinical characteristics, as well as psychological distress (Table 3; details are provided in Table S1 and Table S2). The first step (sociodemographic characteristics) and the second step (clinical characteristics) of the regression model for peaceful acceptance explained 5% and an additional 2% of the variance, respectively, with age being significantly and positively associated with peaceful acceptance. In the third step, psychological distress revealed a significant negative association with peaceful acceptance and accounted for an additional 25% of the variance. Introducing mindfulness and self-compassion in the fourth step explained an additional 4% of the variance, with self-compassion significantly associated with peaceful acceptance. In the fifth step, body image distress revealed a significant negative association, adding another 2% of explained variance. The overall model explained 33% of the variance, *R*^2^ = .33, *F*(1,123) = 6.99, *p* < .001. Psychological distress and body image distress were independently associated with lower levels of peaceful acceptance.

**Table 3.** Hierarchical regression analyses for peaceful acceptance and struggle with illness

	Peaceful acceptance		Struggle with illness	
Variable	*β*	Δ*R*^2^	*β*	Δ*R*^2^
Step 1		.051		.139***
Age	.222*		−.301***	
Sex	.067		−.073	
Education	.066		−.019	
Marital status	.011		−.195*	
Step 2		.019		.018
Age	.205*		−.329***	
Sex	.032		−.078	
Education	.060		−.014	
Marital status	.015		−.169*	
KPS	−.049		−.137	
BMI	.096		−.011	
Months since diagnosis	−.088		−.012	
Step 3		.247***		.277***
Age	.118		−.237**	
Sex	.018		−.064	
Education	.116		−.074	
Marital status	−.076		−.072	
KPS	−.051		−.134	
BMI	.021		.069	
Months since diagnosis	−.086		−.014	
Psychological distress	−.527***		.558***	
Step 4		.043*		.086***
Age	.126		−.249***	
Sex	.008		−.032	
Education	.122		−.080	
Marital status	−.059		−.088	
KPS	−.085		−.090	
BMI	.052		.027	
Months since diagnosis	−.079		−.012	
Psychological distress	−.366***		.376***	
Mindfulness	.115		−.048	
Self-compassion	.186*		−.320***	
Step 5		.024*		.124***
Age	.065		−.109	
Sex	−.001		−.012	
Education	.128		−.096	
Marital status	−.086		−.028	
KPS	−.118		−.016	
BMI	.026		.085	
Months since diagnosis	−.070		−.032	
Psychological distress	−.291**		.206**	
Mindfulness	.153		−.111	
Self-compassion	.143		−.246***	
Body image distress	−.203*		.458***	

BMI, Body Mass Index; KPS, Karnofsky Performance Status.

* *p* < .05; **p < 0.01; ***p < 0.001.

In the regression model for struggle with illness, the sociodemographic characteristics accounted for 14% of the variance (step 1), while the clinical characteristics added another 2% (step 2). In both steps, age and marital status were significantly and negatively associated with struggle with illness. In the third step, psychological distress revealed a significant negative association with struggle with illness, explaining an additional 28% of the variance. Introducing mindfulness and self-compassion in the fourth step explained an additional 9% of the variance, with self-compassion being significantly negatively associated with struggle with illness. In the fifth step, body image distress showed a significant positive association with struggle with illness, accounting for an additional 12% of the variance. The overall model explained 61% of the variance, *R*^2^ = .61, *F*(1,123) = 20.22, *p* < .001. Psychological distress, self-compassion, and body image distress were significant independent factors associated with struggle with illness.

## Discussion

Do mindfulness, self-compassion, and body image distress represent important dimensions for terminally ill cancer patients in peacefully accepting or struggling with their life-limiting condition? This study aimed to address this question, given the limited research on psychological factors that either hinder or promote emotional acceptance of one’s terminal condition.

One of the key findings supporting our hypothesis is that body image distress was uniquely associated with both peaceful acceptance and struggle with illness, even after accounting for psychological distress, sociodemographic, and clinical characteristics. Body image distress may play a central role across the spectrum of various states, those reflecting a struggle with ones’ condition (e.g., feelings of fear, injustice, anger, rage, foreboding), and those reflecting acceptance of one’s condition (e.g., feelings of peace and equanimity). This finding parallels previous studies showing that body image concerns affect both positive and negative functioning in early-stage cancer (Diaz-Frutos et al. 2016; Nikita Rani and Kumar 2022; Paterson et al. 2016; Przezdziecki et al. 2013; Sebri et al. 2024) and metastatic breast cancer patients (McClelland et al. 2015). Our result extends this knowledge to terminally ill cancer patients, showing its relevance to key outcomes in EOL care, namely acceptance and struggle with illness. We interpret this in light of qualitative evidence suggesting that body image distress reflects patients’ lack of control over their bodies and attachment to their former self-image (Vas et al. 2019), which is not a mere problem *per se*, but also a potential vulnerability factor hindering a peaceful acceptance and the letting go of a struggle with one’s condition.

Our correlation analyses showed that terminally ill cancer patients with higher mindfulness and self-compassion tend to report greater acceptance and lower struggle with illness. However, in multivariable analyses, mindfulness was not significantly associated with either peaceful acceptance or struggle with illness. Conversely, self-compassion was strongly associated with lower struggle with illness but did not remain significant for peaceful acceptance. These findings partly support our hypothesis and are consistent with previous studies suggesting that self-compassion may be a stronger predictor of outcomes compared to mindfulness (Neff and Dahm 2015; Svendsen et al. 2020). We indeed expected self-compassion to be especially beneficial at the EOL, as it focuses on painful experiences, self-soothing, and recognizing suffering as part of the shared human experience (Neff and Dahm 2015).

When considering the overall findings, three aspects are noteworthy. First, the strong inverse correlation between peaceful acceptance and struggle with illness is consistent with prior research indicating that as grief states (i.e., disbelief, yearning, anger, and sadness) decrease, acceptance of loss increases (Prigerson and Maciejewski 2008). Second, the same set of predictors explained very different amounts of variance for peaceful acceptance (33%) and struggle with illness (61%). Third, self-compassion was uniquely associated with struggle but not with acceptance. While acceptance and struggle are strongly inversely correlated at a bivariate level, potentially suggesting they represent two sides of the same coin (Prigerson and Maciejewski 2008), multivariate regression analyses point to partially independent clinical pathways with partly distinct predictors. Self-compassion may represent a resource fostering the personal element of the dying role (Emanuel et al. 2007), which helps patients let go of the struggle while approaching death (e.g., less fear, rage, anger, injustice related to the terminal condition), but it may not be as influential in fostering peaceful acceptance (e.g., sense of peace and equanimity). Similarly, body image distress, even if significant on both outcomes, was more strongly associated with struggle with illness than with peaceful acceptance.

These findings, along with the divergent variance explained for peaceful acceptance and struggle with illness, suggest that the psychological factors examined in this study (e.g., body image distress, self-compassion) appear more relevant for explaining struggle with illness. The latter may involve psychological processes related to adjustment to loss (Emanuel et al. 2007), including changes in body and identity (Vas et al. 2019) and the regulation of distressing emotions (e.g., kindness toward one’s suffering; Neff and Dahm 2015, 2015). In contrast, the lower variance explained for peaceful acceptance suggests the involvement of additional factors beyond those examined, potentially linked to dimensions of the personal element of the dying role beyond adjustment to loss, such as the engagement with existential questions and reaching closure with meaningful others (Emanuel et al. 2007). For instance, social/family well-being (i.e., the sense of closeness, emotional support, and effective communication with family and friends about the illness) was significantly correlated with peaceful acceptance but not with struggle with illness (Okamura et al. 2022). Future longitudinal studies could examine the dynamic interplay between struggle with illness and peaceful acceptance, as well as identify differential predictors of the two. Such studies could benefit from further examining the roles of self-compassion and body image distress while also incorporating predictors identified in previous research as contributors to emotional preparedness, another conceptualization of emotional acceptance. Emotional preparedness has been associated with factors reflecting engagement with others, such as social support and patient-family communication about EOL issues (Wen et al. 2023; Wentlandt et al. 2012). Additionally, existential issues such as finding and/or making meaning in the terminally ill’s life may warrant further investigation, as they have been linked to various positive EOL outcomes, including a greater sense of peace (De Vincenzo et al. 2023; Iani et al. 2020; Rosenfeld et al. 2018; Terao and Satoh 2022).

According to our data, terminally ill cancer patients may benefit from a dual-target approach. On the one hand, patients struggling with their condition may benefit from interventions targeting body image concerns, psychological distress, and self-compassion. On the other hand, psychological and body image distress may be targeted to promote or maintain peaceful acceptance. A plethora of research examined the efficacy of psychological interventions on body image in cancer patients and survivors, predominantly focusing on breast cancer (Morales-Sánchez et al. 2021; Sebri et al. 2021; Sebri and Pravettoni 2023). Since quantitative studies with terminally ill patients are very limited, our results emphasize the need for further research, which should also include the development of tailored interventions for body image concerns of the terminally ill. Similarly, self-compassion-based interventions have been examined in various chronic conditions (Mistretta and Davis 2022), with initial evidence suggesting the feasibility, acceptability, and benefits of a meaning-centered intervention integrated with elements of compassion and tailored to the needs of terminally ill cancer patients (Gil et al. 2018). Future studies should examine if these interventions are effective in improving patients’ struggle with their condition and/or fostering peaceful acceptance.

Finally, a relevant implication concerns with the conceptualization of emotional acceptance. Recent research conceptualized emotional acceptance as emotional preparedness for death (Tang et al. 2019, 2020; Wen et al. 2022, 2021, 2023), as measured with the Preparation for EOL subscale of the Quality of Life at the End of Life scale (QUAL-E), which includes patient concerns about becoming a burden, reflection on life regrets, perceptions of the extent to which one’s family is prepared for the patient’s end of life, and fear of dying (Lo et al. 2011; Steinhauser et al. 2004). While it has been proposed that both peacefully accepting and struggling with one’s terminal condition are tapped by two items of QUAL-E’s Preparation subscale (fear of dying and reflection on life regrets; Tang et al. 2019), empirical studies are missing. Future research should investigate if and to what extent the PEACE and preparation-QUAL-E overlap, and if they both reflect elements of an overarching construct. Moreover, according to our findings, future studies investigating differential predictors of acceptance and/or struggle, or tailored interventions may benefit from considering both peaceful acceptance and the struggle with illness as separate outcomes.

These results should be interpreted in light of several limitations. First, the cross-sectional design of the study precludes causal inferences. Second, terminally ill cancer participants were consecutively sampled from a single Hospice, potentially reflecting a specific clinical setting and limit the generalizability of the findings to patients in other settings or those not enrolled in palliative care. While this study focused on patients in palliative care, the findings may be not generalizable to terminally ill patients with similar prognoses who are not receiving hospice or palliative care, as the type and intensity of support likely differ in meaningful ways. Moreover, although we conducted a power analysis confirming that the sample size provides adequate statistical power, future studies with larger and more diverse samples across multiple sites would be beneficial to further enhance generalizability. These issues may be addressed by future longitudinal and multicenter studies. Third, the variability within sample, including differences in cancer type, progression, and individual coping strategies, could influence the results. A larger sample size could provide a more comprehensive understanding of how the examined variables interact across different subgroups of terminally ill cancer patients.

This study highlights the roles of body image distress and self-compassion as significant factors in influencing struggle with illness and/or peaceful acceptance in terminally ill cancer patients. Importantly, our findings suggest that these two outcomes may reflect partly distinct clinical pathways, underpinned by partly different psychological mechanisms. Addressing body image distress and fostering self-compassion could represent targeted intervention strategies to improve patients’ adaptation to terminal illness. Further longitudinal research is essential to elucidate these pathways and evaluate their impact across diverse populations and clinical settings.

## Supporting information

De Vincenzo et al. supplementary material 1 De Vincenzo et al. supplementary material

De Vincenzo et al. supplementary material 2 De Vincenzo et al. supplementary material

## Supplementary material

The supplementary material for this article can be found at https://doi.org/10.1017/S1478951525000094.
