# Supplementary material for: Peaceful acceptance and struggle with terminal cancer: The role of mindfulness, self-compassion, and body image distress
Source: Palliat Support Care. 2025 Mar 14;23:e76. doi: 10.1017/S1478951525000094 (PMC13166399; doi:10.1017/S1478951525000094)
Supplement: De Vincenzo et al. supplementary material 1 — De Vincenzo et al. supplementary material [file S1478951525000094sup001.docx]

| **Table S1.** Hierarchical regression analysis for peaceful acceptance | | | | | | | | | | | |
| --- | --- | --- | --- | --- | --- | --- | --- | --- | --- | --- | --- |
|  | Variable | *B* | 95% CI | | SE | *β* | *t* | Sig. | Adjusted *R*² | SE of the estimate | *R*² change |
|  |  |  | *LL* | *UL* |  |  |  |  |  |  |  |
| Step 1 | (Constant) | 6.893 | 1.716 | 12.069 | 2.617 |  | 2.634 | .009 | .022 | 3.302 | .051 |
|  | Age | .062 | .012 | .112 | .025 | .222 | 2.458 | .015 |  |  |  |
|  | Sex | .446 | -.700 | 1.591 | .579 | .067 | .770 | .443 |  |  |  |
|  | Education | .223 | -.380 | .827 | .305 | .066 | .732 | .466 |  |  |  |
|  | Marital Status | .075 | -1.070 | 1.221 | .579 | .011 | .130 | .897 |  |  |  |
| Step 2 | (Constant) | 6.742 | 0.019 | 13.465 | 3.398 |  | 1.984 | .049 | .019 | 3.307 | .019 |
|  | Age | .057 | .006 | .109 | .026 | .205 | 2.210 | .029 |  |  |  |
|  | Sex | .214 | -.970 | 1.398 | .598 | .032 | .357 | .721 |  |  |  |
|  | Education | .203 | -.403 | .810 | .307 | .060 | .662 | .509 |  |  |  |
|  | Marital Status | .099 | -1.071 | 1.269 | .591 | .015 | .168 | .867 |  |  |  |
|  | KPS | -.024 | -.114 | .065 | .045 | -.049 | -.540 | .590 |  |  |  |
|  | BMI | .077 | -.063 | .218 | .071 | .096 | 1.088 | .279 |  |  |  |
|  | TSD | -.006 | -.019 | .006 | .006 | -.088 | -.997 | .321 |  |  |  |
| Step 3 | (Constant) | 13.020 | 6.948 | 19.093 | 3.068 |  | 4.243 | .000 | .274 | 2.846 | .247 |
|  | Age | .033 | -.012 | .078 | .023 | .118 | 1.457 | .148 |  |  |  |
|  | Sex | .123 | -.896 | 1.143 | .515 | .018 | .239 | .811 |  |  |  |
|  | Education | .392 | -.133 | .917 | .265 | .116 | 1.478 | .142 |  |  |  |
|  | Marital Status | -.508 | -1.530 | .515 | .517 | -.076 | -.982 | .328 |  |  |  |
|  | KPS | -.026 | -.103 | .051 | .039 | -.051 | -.659 | .511 |  |  |  |
|  | BMI | .017 | -.105 | .139 | .062 | .021 | .273 | .786 |  |  |  |
|  | TSD | -.006 | -.017 | .005 | .005 | -.086 | -1.136 | .258 |  |  |  |
|  | PHQ-4 | -.570 | -.737 | -.402 | .084 | -.527 | -6.746 | .000 |  |  |  |
| Step 4 | (Constant) | 6.073 | -1.855 | 14.001 | 4.006 |  | 1.516 | .132 | .309 | 2.776 | .043 |
|  | Age | .035 | -.008 | .079 | .022 | .126 | 1.599 | .112 |  |  |  |
|  | Sex | .052 | -.962 | 1.066 | .512 | .008 | .102 | .919 |  |  |  |
|  | Education | .410 | -.103 | .922 | .259 | .122 | 1.583 | .116 |  |  |  |
|  | Marital Status | -.395 | -1.398 | .609 | .507 | -.059 | -.779 | .438 |  |  |  |
|  | KPS | -.043 | -.119 | .033 | .038 | -.085 | -1.116 | .267 |  |  |  |
|  | BMI | .042 | -.079 | .162 | .061 | .052 | .682 | .496 |  |  |  |
|  | TSD | -.006 | -.016 | .005 | .005 | -.079 | -1.065 | .289 |  |  |  |
|  | PHQ-4 | -.396 | -.606 | -.185 | .106 | -.366 | -3.724 | .000 |  |  |  |
|  | FFMQ-SF | .036 | -.024 | .095 | .030 | .115 | 1.189 | .237 |  |  |  |
|  | SCS-SF | .098 | .008 | .188 | .046 | .186 | 2.145 | .034 |  |  |  |
| Step 5 | (Constant) | 9.193 | 0.897 | 17.489 | 4.191 |  | 2.194 | .030 | .330 | 2.734 | .024 |
|  | Age | .018 | -.028 | .064 | .023 | .065 | .781 | .436 |  |  |  |
|  | Sex | -.007 | -1.007 | .994 | .505 | -.001 | -.013 | .989 |  |  |  |
|  | Education | .433 | -.072 | .938 | .255 | .128 | 1.697 | .092 |  |  |  |
|  | Marital Status | -.573 | -1.574 | .428 | .506 | -.086 | -1.133 | .259 |  |  |  |
|  | KPS | -.059 | -.136 | .017 | .039 | -.118 | -1.540 | .126 |  |  |  |
|  | BMI | .021 | -.099 | .141 | .061 | .026 | .342 | .733 |  |  |  |
|  | TSD | -.005 | -.015 | .005 | .005 | -.070 | -.957 | .341 |  |  |  |
|  | PHQ-4 | -.315 | -.534 | -.095 | .111 | -.291 | -2.839 | .005 |  |  |  |
|  | FFMQ-SF | .044 | -.015 | .103 | .030 | .143 | 1.487 | .140 |  |  |  |
|  | SCS-SF | .081 | -.010 | .171 | .046 | .153 | 1.771 | .079 |  |  |  |
|  | BIS | -.087 | -.166 | -.009 | .040 | -.203 | -2.204 | .029 |  |  |  |
| *Note*. BIS = Body Image Scale; BMI = Body Mass Index; FFMQ-SF = Five Facet Mindfulness Questionnaire-Short Form; KPS = Karnofsky Performance Status; PHQ-4 = Patient Health Questionnaire-4; SCS-SF = Self-Compassion Scale-Short Form; TSD = Time Since Diagnosis (months). | | | | | | | | | | | |
