# Supplementary material for: Peaceful acceptance and struggle with terminal cancer: The role of mindfulness, self-compassion, and body image distress
Source: Palliat Support Care. 2025 Mar 14;23:e76. doi: 10.1017/S1478951525000094 (PMC13166399; doi:10.1017/S1478951525000094)
Supplement: De Vincenzo et al. supplementary material 2 — De Vincenzo et al. supplementary material [file S1478951525000094sup002.docx]

| **Table S2.** Hierarchical regression analysis for struggle with illness | | | | | | | | | | | |
| --- | --- | --- | --- | --- | --- | --- | --- | --- | --- | --- | --- |
|  | Variable | *B* | 95% CI | | SE | *β* | *t* | Sig. | Adjusted *R*² | SE of the estimate | *R*² change |
|  |  |  | *LL* | *UL* |  |  |  |  |  |  |  |
| Step 1 | (Constant) | 30.181 | 23.432 | 36.929 | 3.411 |  | 8.847 | .000 | .113 | 4.305 | .139 |
|  | Age | -.115 | -.180 | -.050 | .033 | -.301 | -3.491 | .001 |  |  |  |
|  | Sex | -.665 | -2.158 | .829 | .755 | -.073 | -.881 | .380 |  |  |  |
|  | Education | -.088 | -.875 | .699 | .398 | -.019 | -.222 | .825 |  |  |  |
|  | Marital Status | -1.780 | -3.274 | -0.287 | .755 | -.195 | -2.358 | .020 |  |  |  |
| Step 2 | (Constant) | 34.091 | 25.329 | 42.853 | 4.428 |  | 7.699 | .000 | .111 | 4.309 | .018 |
|  | Age | -.126 | -.193 | -.059 | .034 | -.329 | -3.727 | .000 |  |  |  |
|  | Sex | -.714 | -2.257 | .829 | .780 | -.078 | -.916 | .361 |  |  |  |
|  | Education | -.067 | -.857 | .724 | .399 | -.014 | -.167 | .868 |  |  |  |
|  | Marital Status | -1.540 | -3.065 | -0.015 | .771 | -.169 | -1.999 | .048 |  |  |  |
|  | KPS | -.094 | -.210 | .023 | .059 | -.137 | -1.594 | .113 |  |  |  |
|  | BMI | -.012 | -.195 | .171 | .093 | -.011 | -.129 | .897 |  |  |  |
|  | TSD | -.001 | -.017 | .015 | .008 | -.012 | -.145 | .885 |  |  |  |
| Step 3 | (Constant) | 24.989 | 17.422 | 32.555 | 3.823 |  | 6.536 | .000 | .398 | 3.546 | .277 |
|  | Age | -.091 | -.146 | -.035 | .028 | -.237 | -3.217 | .002 |  |  |  |
|  | Sex | -.583 | -1.853 | .687 | .642 | -.064 | -.908 | .366 |  |  |  |
|  | Education | -.341 | -.995 | .313 | .331 | -.074 | -1.031 | .304 |  |  |  |
|  | Marital Status | -.660 | -1.934 | .614 | .644 | -.072 | -1.025 | .307 |  |  |  |
|  | KPS | -.092 | -.188 | .004 | .048 | -.134 | -1.901 | .060 |  |  |  |
|  | BMI | .076 | -.077 | .228 | .077 | .069 | .983 | .327 |  |  |  |
|  | TSD | -.001 | -.015 | .012 | .007 | -.014 | -.202 | .840 |  |  |  |
|  | PHQ-4 | .826 | .618 | 1.034 | .105 | .558 | 7.848 | .000 |  |  |  |
| Step 4 | (Constant) | 35.853 | 26.448 | 45.257 | 4.752 |  | 7.545 | .000 | .481 | 3.293 | .086 |
|  | Age | -.095 | -.147 | -.043 | .026 | -.249 | -3.638 | .000 |  |  |  |
|  | Sex | -.292 | -1.495 | .912 | .608 | -.032 | -.480 | .632 |  |  |  |
|  | Education | -.371 | -.979 | .237 | .307 | -.080 | -1.208 | .229 |  |  |  |
|  | Marital Status | -.808 | -1.998 | .382 | .601 | -.088 | -1.344 | .181 |  |  |  |
|  | KPS | -.062 | -.152 | .028 | .046 | -.090 | -1.360 | .176 |  |  |  |
|  | BMI | .029 | -.114 | .172 | .072 | .027 | .405 | .686 |  |  |  |
|  | TSD | -.001 | -.014 | .011 | .006 | -.012 | -.185 | .854 |  |  |  |
|  | PHQ-4 | .556 | .306 | .805 | .126 | .376 | 4.408 | .000 |  |  |  |
|  | FFMQ-SF | -.020 | -.091 | .050 | .036 | -.048 | -.575 | .566 |  |  |  |
|  | SCS-SF | -.230 | -.337 | -.123 | .054 | -.320 | -4.258 | .000 |  |  |  |
| Step 5 | (Constant) | 26.194 | 17.556 | 34.833 | 4.364 |  | 6.002 | .000 | .612 | 2.847 | .124 |
|  | Age | -.042 | -.089 | .006 | .024 | -.109 | -1.741 | .084 |  |  |  |
|  | Sex | -.110 | -1.151 | .932 | .526 | -.012 | -.208 | .835 |  |  |  |
|  | Education | -.443 | -.969 | .082 | .266 | -.096 | -1.669 | .098 |  |  |  |
|  | Marital Status | -.257 | -1.299 | .786 | .527 | -.028 | -.487 | .627 |  |  |  |
|  | KPS | -.011 | -.090 | .069 | .040 | -.016 | -.270 | .788 |  |  |  |
|  | BMI | .094 | -.031 | .219 | .063 | .085 | 1.482 | .141 |  |  |  |
|  | TSD | -.003 | -.014 | .008 | .005 | -.032 | -.579 | .564 |  |  |  |
|  | PHQ-4 | .305 | .077 | .534 | .115 | .206 | 2.645 | .009 |  |  |  |
|  | FFMQ-SF | -.047 | -.108 | .014 | .031 | -.111 | -1.521 | .131 |  |  |  |
|  | SCS-SF | -.177 | -.271 | -.083 | .047 | -.246 | -3.737 | .000 |  |  |  |
|  | BIS | .270 | .189 | .352 | .041 | .458 | 6.552 | .000 |  |  |  |
| *Note*. BIS = Body Image Scale; BMI = Body Mass Index; FFMQ-SF = Five Facet Mindfulness Questionnaire-Short Form; KPS = Karnofsky Performance Status; PHQ-4 = Patient Health Questionnaire-4; SCS-SF = Self-Compassion Scale-Short Form; TSD = Time Since Diagnosis (months). | | | | | | | | | | |  |
